# Acute Diarrheas among French Soldiers in Djibouti

**DOI:** 10.4269/ajtmh.2011.10-0587a

**Published:** 2011-01-05

**Authors:** Thierry Coton

**Affiliations:** Service de Pathologie Digestive; Hôpital d'Instruction des Armées Laveran; Marseille Cedex 13, France; E-mail: thierrycoton@msn.com

Dear Sir:

I read with great interest the article published by Ollivier and others^1^ about gastrointestinal illnesses among French Forces deployed to Djibouti. It offers a panorama of infectious diarrheas occurring in French soldiers deployed in this East African country and points out the high incidence rate of these affections even compared with Western Africa.

I worked in the Medicine Unit of the French military hospital in Djibouti between 2005 and 2007. Infectious diarrheas is a sanitary problem among military communities, especially in Djibouti, as stated in this article.^1^ I also agree with their results on the annual repartition of cases. In our unit, we observed an increased rate of acute diarrheas after the end of September, which corresponds to the beginning of the cool season and the reemergence of many flies, which have disappeared before because of the high temperatures during previous months. The first weeks of the stay in Djibouti are also marked for the huge majority of people by transient stools modifications and upper respiratory tract infections thought to correspond to new bacterial and viral environment adaptation.

However, this work avoids speaking about minor salmonellosis and especially giardiasis, which constitute locally two main etiologies of acute, subacute, and chronic diarrheas in my Djiboutian experience. Giardiasis was a cause of acute non-febrile diarrhea with a trend to chronic evolution and its association with amebiasis, salmonellosis and shigellosis was not uncommon. Giardiasis diagnosis was not easy in Djibouti because only stool smear direct examination was disponible in our laboratory. Duodenal biopsies realized during upper digestive tract endoscopy were sent to France for histological and parasitological examinations in some cases of chronic diarrhea. Therefore, this parasitological diarrhea might be frequently missed. In clinical practice, at the beginning, we used to prescribe metronidazole presumptively as a second-line therapy after first-line treatment failure and later, we associated metronidazole to fluoroquinolone as first-line treatment of each case of acute diarrhea even if initially febrile. Metronidazole was also used as systematic first-line therapy of each case of chronic diarrhea.

Therefore, it would be of great interest to add giardiasis in French soldiers' health surveillance because it is not rare in Djibouti. It can also chronicize or induce chronic intestinal functional disorders,^2^ which could invalidate soldiers during their mission, especially when of short duration.
